# Phenotypic, molecular and biochemical evaluation of somatic hybrids between *Solanum tuberosum* and *S. bulbocastanum*

**DOI:** 10.1038/s41598-022-08424-5

**Published:** 2022-03-16

**Authors:** Petr Sedlák, Vladimíra Sedláková, Jakub Vašek, Dukagjin Zeka, Daniela Čílová, Martina Melounová, Matyáš Orsák, Jaroslava Domkářová, Petr Doležal, Pavel Vejl

**Affiliations:** 1 Department of Genetics and Breeding, Faculty of Agrobiology, Food and Natural Resources, Czech University of Life Sciences Prague, Kamýcká 129, 16500 Prague 6 Suchdol, Czech Republic; 2 Department of Crops, University of Prishtina, Str. “George Bush” 31, 10 000 Pristina, Republic of Kosovo; 3 Department of Chemistry, Faculty of Agrobiology, Food and Natural Resources, Czech University of Life Sciences Prague, Kamýcká 129, 16500 Prague 6 Suchdol, Czech Republic; 4 Department of Genetic Resources, Potato Research Institute Havlíčkův Brod, Ltd., Dobrovského 2366, 58001 Havlíčkův Brod, Czech Republic; 5 Department of Potato Protection, Potato Research Institute Havlíčkův Brod, Ltd., Dobrovského 2366, 58001 Havlíčkův Brod, Czech Republic

**Keywords:** Plant breeding, Agricultural genetics

## Abstract

Somatic hybridization has been frequently used to overcome sexual incompatibility between potato and its secondary germplasm. The primary objective of this study was to produce and evaluate somatic hybrids of *Solanum tuberosum* (*Stub*) and *S. bulbocastanum* (*Sblb*) for breeding purposes. In 2007, 23 somatic hybrids were produced using an electrofusion of mesophyll protoplasts of diploid (2n = 2x = 24) potato line *Stub*DH165 and *S. bulbocastanum* PI24351 (*Sblb*66). Phenotype of somatic hybrids in field conditions were evaluated, together with constitution and stability of 30 nuclear (ncSSR) and 27 cytoplasmic (cpSSR) microsatellite markers and content of main glycoalkaloids. All somatic hybrids had very high field resistance against late blight, but the plants were infertile: the viability of pollen grains insignificantly varied between 0.58 and 8.97%. A significant somaclonal variation was observed in terms of the morphology of plants, the date of emergence, the quantity of harvested tubers, the content of glycoalkaloids in foliage, and nuclear microsatellite markers (ncSSR). The analysis of ncSSR identified five distinct genotypes of hybrids partly associated with phenotype variations. The process of somatic hybridization with regeneration of shoots was identified as the most likely source of somaclonal variation because the ncSSR genotypes of hybrids, which were maintained in vitro, remained stable for more than 10 years. The infertile somatic hybrids have no practical breeding potential, but they are considered very suitable for advanced studies of the differential expression of genes in the pathways linked to dormancy of tubers and synthesis of glycoalkaloids.

## Introduction

Potato (*Solanum tuberosum* ssp. *tuberosum* L.), the third most important food crop worldwide, following rice and wheat^1^, belongs to the genus *Solanum* section *Petota*., that includes four cultivated species^2^ and around 100 wild tuber-bearing species^3^. The latest complex analyses of DNA sorted the species into four clades^4^, which have helped in understanding phylogenetic, geographical, and genetic relationships potentially influencing the utilization of germplasm in breeding. In particular, the wild species have been frequently used in potato breeding programs, as they are genetic resources for many highly valued resistance genes. Interspecific hybridization, used for introgression of the genes to the potato germplasm, is frequently disturbed or prevented by different prezygotic and postzygotic barriers^5^, which can be overcome through somatic hybridization^6–8^. In the last decade, *S. bulbocastanum* Dun. (sect. *Petota*, 2n = 2x = 24, 1EBN, clade 2), which is sexually incompatible with cultivated potato^9^ and has a very high resistance to late blight caused by *Phytophthora infestans* (Mont.) de Bary^10^, has been frequently used for somatic hybridization. In this regard, the genomes of potato and *S. bulbocastanum*, respectively reported previously as A and B genomes^4,11,12^, have been studied. Significant structural differences in *Stub* + *Sblb* somatic hybrids were reported^7^ and documented by typical meiotic anomalies, such as loops in paired “A and B” chromosomes at pachytene, dicentric chromatid bridges at M-I anaphase, various meiotic spindle orientations and various numbers of resulting meiotic cells. Subsequently, the 2S and 8S chromosomal regions in the B genome distinct from that found in the A genome were described using a DArT (Diversity Arrays Technology) marker-based linkage map^9^.

Breeding approaches in vitro, including somatic hybridization, represent a potential resource of somaclonal variation^13,14^, which likely extends diversity in clonal progenies. Practical experiences and the value of somaclonal variation for potato breeding^15^, and the most prominent genetic conditions of somaclonal variation^16^ were reviewed. The studies mainly referred to mutations/genetic rearrangements in somatic cells and their insufficient repair under the effect of specific cultivation conditions, especially by cytokinins used for regeneration of plants in cell cultures. This could also influence the epigenetic aspects of somaclonal variation, such as DNA methylation and activation of various DNA elements across the genome. Epigenetic aspects, which were summarized and discussed previously^17^, were identified as less stable between regenerated plants and their progeny.

The detection of these genetic changes requires extensive molecular genetic analyses. Larger rearrangements are potentially detectable using various explorative methods of DNA analysis. In the past, potato genetic research produced several panels of SSR (simple sequence repeats) markers detecting abundant polymorphisms of microsatellites^13,18–22^. They are generally considered strong in terms of the range of polymorphisms and regular dispersion across genomes. Consequently, they have been used for the identification of potato genetic resources^21^. Microsatellites can also be considered very useful for the study of the genetic instability of plants because their rearrangements are tightly associated with dysfunctions in DNA mismatch repair systems^23^. In addition to microsatellite markers, DArT assays were developed and used specially for the extensive assessment of variations in different *Solanum* genomes^9^ and in interspecific somatic hybrids^24^.

The primary objective of this research was to produce somatic hybrids of 2n = 2x = 24 *S. tuberosum* ssp. *tuberosum* (*Stub*) and *S. bulbocastanum* (*Sblb*) PI24351 resistant to late blight and evaluate their potential for breeding. Being based on the hypothesis that phenotype differences should associate with changes of microsatellite loci, the article informs about findings of our long-term study of somaclonal variation observed within the somatic hybrids.

## Material and methods

### Plant material

The plants obtained and analyzed in the presented study are introduced in Table 1. Parental genotypes chosen for somatic hybridization were kindly provided by the gene bank in vitro of the Potato Research Institute Havlíčkův Brod Ltd. According to the information from the provider, the parental 2n = 2x = 24 genotype *Stub*DH165 (*Solanum tuberosum* ssp. *tuberosum*; *Stub*) was induced by inter-specific hybridization of 2n = 4x = 48 potato variety ‘Apta’ with 2n = 2x = 24 inducer of diploidy *S. phureja* Juz. & Bukasov (*Sphu*) 85/16. The parental genotype *Sblb*66 (*S. bulbocastanum*; *Sblb*) was chosen from population PI243510 as a donor of the *Rpi-blb1* gene.

**Table 1:** Characteristics of parents and somatic hybrids.

Ref. number	Genotype code	GRIN identification number	Origin (pedigree)	NcSSR genotype	Ploidy
1	*Stub*DH165	07S0500005	*Stub* ‘Apta’ x *Sphu*	NA	2n = 2x = 24
2	*Sblb*66	07S0300335	*Sblb* PI243510	NA	2n = 2x = 24
3	REG28F	NA	Somatic hybrid, callus 10	A	2n = 4x = 48
4	REG30F	07S0200434	Somatic hybrid, callus 10	B	2n = 4x = 48
5	REG32F	07S0200435	Somatic hybrid, callus 10	B	2n = 4x = 48
6	REG38F	07S0200436	Somatic hybrid, callus 10	B	2n = 4x = 48
7	REG39F	NA	Somatic hybrid, callus 10	A	2n = 4x = 48
8	REG40F	NA	Somatic hybrid, callus 10	A	2n = 4x = 48
9	REG42F	NA	Somatic hybrid, callus 10	B	2n = 4x = 48
10	REG70F	NA	Somatic hybrid, callus 10	A	2n = 4x = 48
11	REG71F	NA	Somatic hybrid, callus 10	A	2n = 4x = 48
12	REG72F	NA	Somatic hybrid, callus 10	B	2n = 4x = 48
13	REG34F	07S0200437	Somatic hybrid, callus 11	C	2n = 4x = 48
14	REG44F	07S0200438	Somatic hybrid, callus 11	C	2n = 4x = 48
15	REG45F	NA	Somatic hybrid, callus 11	C	2n = 4x = 48
16	REG46F	07S0200439	Somatic hybrid, callus 11	C	2n = 4x = 48
17	REG48F	NA	Somatic hybrid, callus 11	C	2n = 4x = 48
18	REG49F	NA	Somatic hybrid, callus 11	A	2n = 4x = 48
19	REG50F	NA	Somatic hybrid, callus 11	A	2n = 4x = 48
20	REG51F	NA	Somatic hybrid, callus 11	A	2n = 4x = 48
21	REG67F	NA	Somatic hybrid, callus 11	C	2n = 4x = 48
22	REG68F	NA	Somatic hybrid, callus 11	A	2n = 4x = 48
23	REG69F	NA	Somatic hybrid, callus 11	C	2n = 4x = 48
24	REG35F	07S0200441	Somatic hybrid, callus 12	D	2n = 4x = 48
25	REG52F	07S0200442	Somatic hybrid, callus 12	E	2n = 4x = 48

More associated data are available under accession numbers of GRIN database (http://grinczech.vurv.cz/gringlobal/search.aspx). The ncSSR genotype (A–E) refers on specific set of microsatellite alleles further characterized in Table 2. *Sblb—Solanum bulbocastanum*, *Stub*—*S. tuberosum* ssp. *tuberosum*; *Sphu*—*S. phureja.*

### Somatic hybridization

The protoplasts were isolated based on methods by Carlberg et al*.*^25^ and fused by Electro Cell Manipulator BTX 2001 (BTX, USA) in a glass microslide with rectangular electrodes with a 3.2 mm gap. The protoplasts were arranged in chains using an impulse of AC (55 V cm^−1^, 12–15 s), fused by an impulse of DC (2100 V cm^−1^, 60 μs), and recovered post-fusion (55 V cm^−1^, 20 s). Media for electrofusion, cultivation, and regeneration of calli and shoots and the remaining cultivation conditions were as reported by Cheng and Saunders^26^. After rooting, the maintained plants were sub-cultivated monthly on original Murashige and Skoog^27^ medium containing vitamins (Duchefa, Netherlands), supplemented with 6.5% agar and 30 g of sucrose. A representative selection of the hybrids is available in the Potato Gene Bank in vitro of the Potato Research Institute Havlíčkův Brod Ltd.

### Ploidy detection

The ploidy of regenerated plants was detected using flow cytometry. Approximately 1 cm^2^ of leaf tissue was cut with a razor blade in 0.5 mL of Otto I buffer (0.1 M citric acid monohydrate and 0.5% (v/v) Tween 20), and the suspension was filtered through a 42 μm nylon mesh. The filtrate was incubated for 10 min at room temperature and 1 mL of Otto II buffer (0.4 M Na_2_HPO_4_.12H_2_O), 4,6-diamidino-2-phenylindole (4 μg mL^−1^) and β-mercaptoethanol (2 μL mL^−1^) were added. The fluorescence intensity was measured using a Partec PAS analyzer (Germany).

### Field experiments and phenotype evaluation

The morphological characteristics of foliage and tubers were evaluated according to Vidner et al.^28^, pollen viability as in Wang et al.^29^, and resistance against late blight of somatic hybrids was evaluated in field and laboratory^30^ experiments. Also, the content of glycoalkaloids (α-chaconine and α-solanine) in mg kg^−1^ of dry matter was quantified in both foliage and tubers. Detailed conditions of the field, laboratory and biochemical evaluations are presented in Supplementary file S8.

### Molecular analysis of DNA

DNA of all samples were isolated twice, in 2009 and 2020, using the DNeasy Plant Mini Kit (Qiagen, Germany) and gradually analyzed using 57 SSR markers; 27 cytoplasmic microsatellites (cpSSR) and 30 nuclear microsatellites (ncSSR) were divided into 13 multiplexes. The positioning of ncSSR loci is presented in Supplementary Figure S1 designed using R-software^31^. The final set of markers comprised 23 plastid SSR markers by Bryan et al*.*^32^, 4 mitochondrial SSR markers by Hosaka and Sanetomo^33^, 24 ncSSR from the potato genetic identity kit by Ghislain et al*.*^21^ and 6 ncSSR markers related to the tuber starch content^18,19,22^. The obtained PCR products were analyzed by capillary electrophoresis ABI PRISM 310 (Applied Biosystems, USA). Length polymorphisms were detected using size standard GeneScan LIZ600 (Applied Biosystems, USA) by GeneMapper v 4.1 software (Applied Biosystems, USA). In addition to cpSSR markers, the type of cytoplasm was identified for all genotypes using the original methodology by Hosaka and Sanetomo^34^. All methodological details of DNA analyses and needed references are presented in Supplementary Tables S1-S4.

### Statistical evaluation

Quantitative data collected during field evaluations and glycoalkaloids content were statistically evaluated using ANOVA and Tukey’s post-hoc analysis. The variability of morphological descriptors was analyzed by Kruskal–Wallis ANOVA. Statistical evaluations were performed on the standard level of significance (α = 0.05) using Dell Statistica Software (Dell Inc, USA).

Allelic data from SSR analysis were processed and sorted in MS Excel 2016. The matrix of dissimilarities was calculated directly from allelic data based on simple matching using the Darwin 6 software^35^. The dendrogram was then calculated using the method of neighbor joining with 30,000 bootstraps and designed within the same software.

### Ethics approval

The study complied with relevant institutional, national, and international guidelines and legislation.

## Results

### Somatic hybridization

The methods of isolation, fusion and regeneration of protoplasts resulted in 3042 pieces of microcallus (approximately 0.5 mm) which were individually plated. Subsequently, 595 calli were grown, but only three of them (calli no. 10, 11 and 12) regenerated into 40 independent shoots. Once the shoots were rooted, 23 green, well-growing plants were obtained. Except REG52E, all adapted well ex vitro. All plants were identified as tetraploid (2n = 4x = 48) somatic hybrids using flow cytometry and subsequent analyses of DNA. Another 17 shoots were obtained, 11 being green and 6 exhibiting albinism, which gradually died being unable to form roots.

### Genetic variation in somatic hybrids

**Figure 1:**
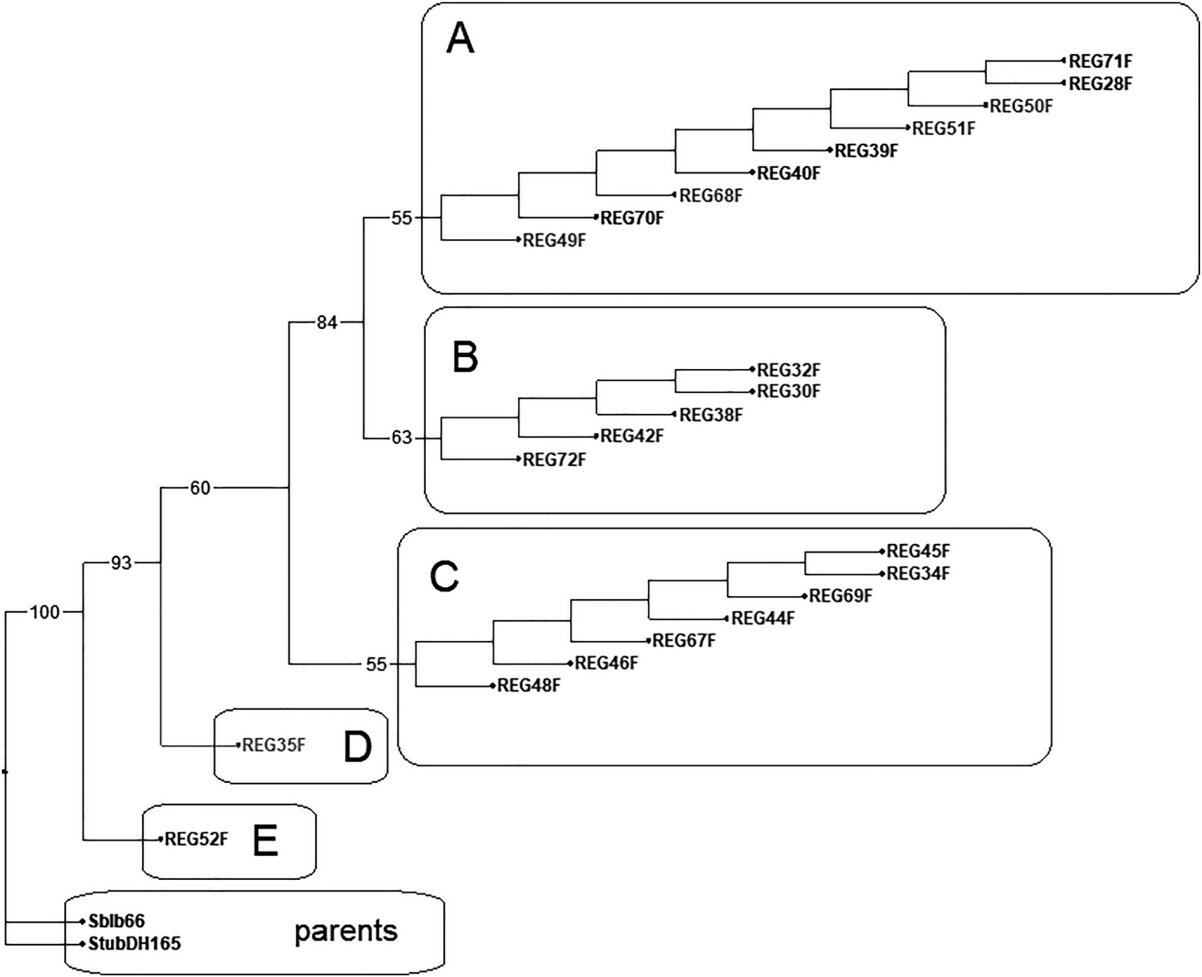
Genetic distances in the ncSSR of somatic hybrids indicates somaclonal variation dividing the somatic hybrids into five distanced groups. Designed in Darwin 6 software^35^, processed in Zoner Photo Studio 14.

Analysis of 30 ncSSR loci detected 65 alleles specific for parents (*Stub*/*Sblb*-specific alleles). In somatic hybrids generally, the appearance of a new 161 bp allele in locus STI028 on chromosome 11 was detected as a result of deletion in *Sblb* specific 164 bp allele. Also, a loss of 3–5 parental alleles to benefit the null alleles was detected in somatic hybrids. Comparing the ncSSR allelic patterns in somatic hybrids, 5 different ncSSR genotypes (A–E) were identified (Fig. 1). The changes in counts of alleles in ncSSR genotypes are specified in Table 2. The most frequent ncSSR genotype A occurred equally in plants regenerated from calli 10 and 11 (Table 1, Fig. 1), whereas the remaining genotypes originated more specifically: genotype B was related to callus 10, genotype C to callus 11 and genotypes D and E to callus 12. The highest number of mutations, detected in E genotype of hybrid REG52F, was coupled with an inability of the hybrid to grow ex vitro and prevented subsequent ex vitro evaluations.

**Table 2:** Incidence of *Stub*DH-specific, *Sblb*-specific, combined, new and null alleles of ncSSR markers in parents and in groups of somatic hybrids classified according the specific ncSSR genotype (A–E).

	*Stub*DH alleles	*Sblb* alleles	Combined alleles	New alleles	Null alleles
*Stub*DH165	42	NA	5	NA	0
*Sblb*66	NA	18	5	NA	12
ncSSR genotype A	41	16	5	1	15
ncSSR genotype B	40	16	5	1	16
ncSSR genotype C	42	16	5	1	14
ncSSR genotype D	41	16	5	1	15
ncSSR genotype E	41	14	5	1	17

The semiautonomous organelles were inherited from *Stub*DH165, with the identity of plastids and mitochondria confirmed by cpSSR markers. Using the rapid method for potato cytoplasm characterization according to Hosaka and Sanetomo^34^, the D type of cytoplasm was detected in both *Stub*DH165 and all somatic hybrids. The W type was detected only in parent *Sblb*66. Allelic data confirming these results are presented in Supplementary data (Supplementary Tables S5 and S6).

### Characteristics of somatic hybrids and somaclonal variation in the field tests

**Figure 2:**
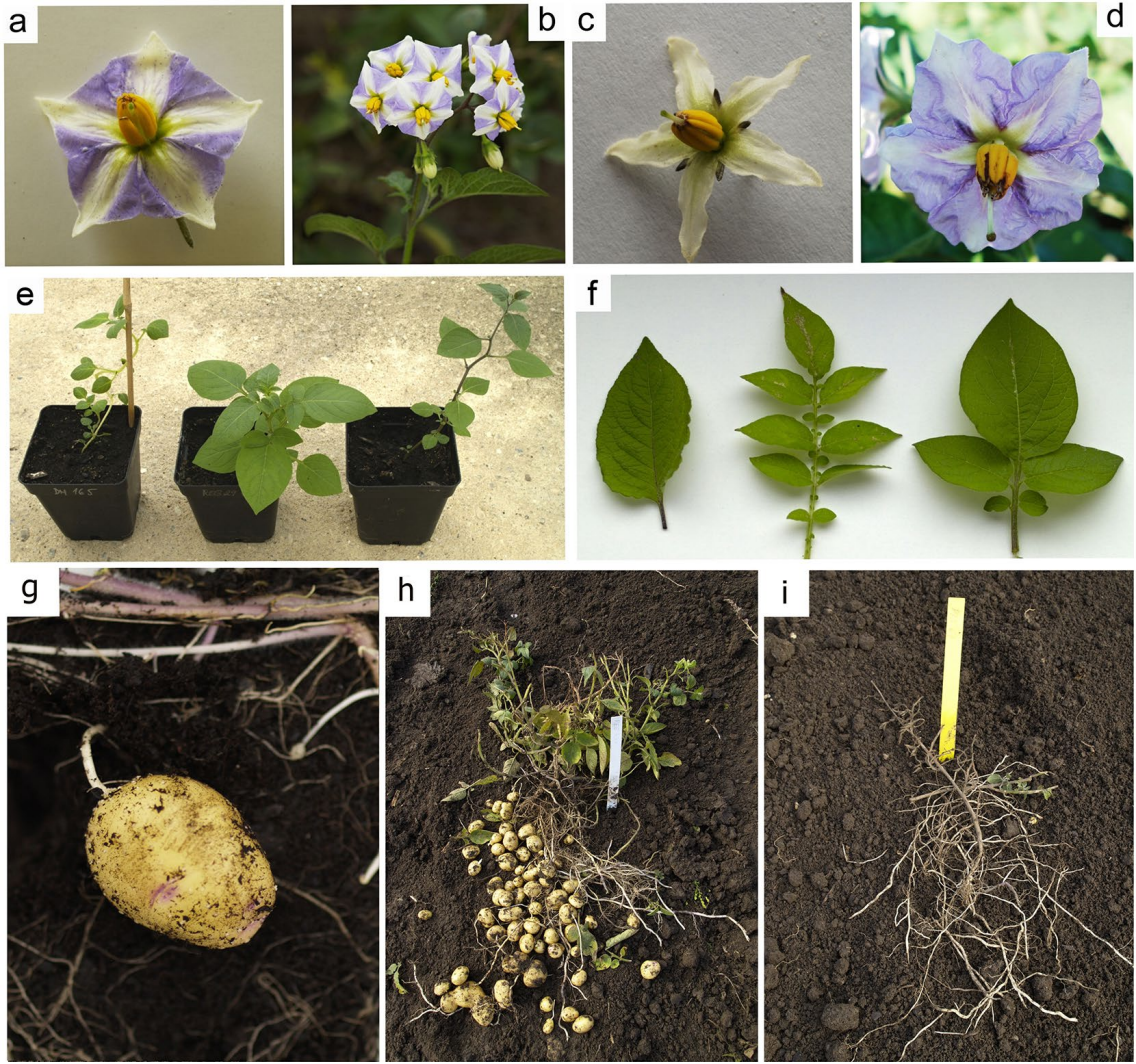
Flowers of somatic hybrids (**a**, **b**) compared to *Sblb*66 (**c**) and *Stub* cv. ‘Apta’ (**d**), intermediate characteristics of somatic hybrids visible in habitus of plants (**e**—from the left: *Stub*DH165, somatic hybrid, *Sblb*66) and leaves (**f**—from the left: *Sblb*66*, Stub*DH165, somatic hybrid), example of tuber of somatic hybrids (**g**), entire plant of somatic hybrid (**h**), entire plant of *Sblb*66 (**i**). Processed in Zoner Photo Studio 14.

Compared to parental species, field tests showed that all somatic hybrids had a more intensive growth and sturdy habitus with a generally intermediate appearance in morphology, particularly regarding characteristics of stems, leaves, and flowers (Fig. 2). For example, from the combination of characteristics of the flower crown, it is visible that the “Rotate” phenotype is identical with *Stub* and can be considered dominant over the “Stellar” phenotype of *Sblb*. Similarly, the shape and size of the cream-colored “inner star” in flowers of somatic hybrids is identical to *Sblb*, which, together with other noticeable characteristics, morphologically confirms the pedigree of hybrids. All the hybrids were generally considered very late in terms of initiation of tubers. The tubers were induced at the start of September (short day adaptation) as with *Sblb*. Flowering started in mid-June and persisted to the first frosts in October.

Somaclonal variation was observed in the phenological development of plants, when the individuals with the ncSSR genotype A, tended to be earlier in terms of the date of emergence and flowering start (Supplementary Figure S2). Conversely, a longer dormancy and later date of emergence were typical for REG35F (ncSSR genotype D). Significant phenotype differences were also observed in six morphological characteristics, in the number and weight of tubers, and in contents of glycoalkaloids in foliage (Table 3 and Supplementary Table S7). The tendency to be different in all characteristics was noticeable for clones having genotype A and D. The results were compared only with *Sblb*66, since *Stub*DH165 failed to grow effectively in field conditions and did not provide reliable data.

**Table 3:** The comparison of the ncSSR genotype groups (A, B, C, D) of somatic hybrids with parent *Sblb66* in characteristics showing significant somaclonal variation (*P* < 0.05).

Characteristics	Genotype				
	*Sblb66*	A	B	C	D
**Morphological descriptors**					
Uprightness of the stem	9.0^b^	5.0^ab^	3.0^a^	4.9^ab^	5.0^ab^
Stem thickness	4.4^a^	6.0^ab^	6.25^ab^	6.1^ab^	6.50^b^
Crown diameter	1.2^a^	4.0^ab^	5.75^ab^	5.2^ab^	7.58^b^
Manifestation of inflorescence	3.4^a^	6.5^ab^	7.63^ab^	6.8^ab^	8.17^b^
Tuber shape	9.0^b^	5.5^a^	5.25^a^	6.0^ab^	5.83^a^
Color of the flesh	7.0^b^	4.5^a^	6.50^ab^	5.0^ab^	4.67^a^
**Yield properties**					
Number of tubers per plot	40.0^b^	160.75^a^	115.57^a^	139.91^a^	135.0^a^
Weight of tubers per plot (kg)	0.05^a^	4.55^ cd^	2.29^b^	5.71^d^	2.38^bc^
Number of tubers over 3 cm per plot	NA	80.00^ab^	54.14^a^	90.46^b^	57.50^ab^
Weight of tubers over 3 cm per plot (kg)	NA	3.55^ab^	1.73^a^	5.20^b^	1.70^a^
**Content of glycoalkaloids in dry matter**					
Chaconine in tubers (mg kg^−1^)	18.80^a^	481.10^b^	549.68^b^	478.06^b^	594.63^b^
Solanine in tubers (mg kg^−1^)	15.15^a^	490.44^b^	560.43^b^	512.46^b^	524.03^b^
Total glycoalkaloids in tubers (mg kg^−1^)	33.95^a^	971.54^b^	1110.11^b^	990.52^b^	1118.65^b^
Chaconine in foliage (mg kg^−1^)	35.45^a^	1022.09^bc^	567.37^ab^	1161.08^c^	2694.67^d^
Solanine in foliage (mg kg^−1^)	23.75^a^	288.57^bc^	161.99^ab^	276.72^c^	784.30^d^
Total glycoalkaloids in foliage (mg kg^−1^)	59.20^a^	1310.67^bc^	729.37^ab^	1437.79^c^	3478.97^d^

Footnotes (a, b… etc.) indicate placement of mean values into homogeneous groups.

The significantly highest yield potential expressed as the number and weight of tubers above 3 cm, equivalent to approximately 5 tons of marketable tubers per hectare, was detected in the group of hybrids having the ncSSR genotype C; the plants from groups B and D were the least productive.

The values of α-chaconine and α-solanine in tubers and foliage of *Sblb*66 were generally very low. Evaluating specifically the α-solanine content in *Sblb*66, two significant extra peaks referring to relatively low concentrations of two unfamiliar glycoalkaloids were detected. Conversely, the total content of glycoalkaloids in tubers of somatic hybrids was significantly higher. Considering a ratio of 1:1 of estimated contents of solanine to chaconine in tubers, the content of solanine in the foliage in comparison to chaconine was approximately four times lower. With the average content of dry matter at a level of 24% of fresh tubers, the total content of alkaloids in tubers exceeded the admissible norm for safe consumption (200 mg kg^−1^ of fresh tubers). Moreover, the tubers were not suitable for consumption based on the subsequent strong bitter taste. The genotype REG35E (12D) showed the significantly highest contents of glycoalkaloids in foliage, which was associated with the loss of *Stub* 126 bp allele of the marker STI0014 positioned on the chromosome 09.

### Resistance to late blight and pollen viability

All somatic hybrids showed a high late blight resistance in both laboratory and field experiments, although it was not as strong as in *Sblb*66. In the field experiments, very high resistance was detected without any symptoms of disease in either leaves and stems. In laboratory experiments, however, the majority of somatic hybrids showed medium to high resistance accompanied by the saprophytic growth and occasionally very weak sporulation of the pathogen in necrotic tissues of leaves. This behavior of pathogen was linked to the environment of the Petri dish. In contrast, the susceptible progenitor *Stub*DH165 allowed the pathogen to produce spores in the whole leaf area and then destructions of leaf tissues were observed.

All the somatic hybrids were infertile, even though they produced a considerable amount of pollen grains and visible eggs in ovaries for the entirety of the growing season. Self-pollinations and artificial crossing between somatic hybrids, backcrosses, or crosses with *Stub* varieties did not result in any berry or seed, and all concerned flowers of somatic hybrids fell within the three days following pollination. The viability of the pollen tested using the methodology by Wang et al*.*^29^ varied insignificantly within somatic hybrids in a range from 0.58 to 8.97%. In contrast, the standards (variety ‘Apta’ and *Sblb*66) showed 74% viability which differed significantly from the somatic hybrids (*P* < 0.01). These genotypes were able to produce vigorous seeds from open pollination.

## Discussion

Yielding 23 vital somatic hybrids, the *Stub*DH165 was successfully used in somatic hybridization with *Sblb*66. The infertility of somatic hybrids is the most challenging problem and is explainable by considering the evolutionary distinctness of *Stub* and *Sblb* genomes^4^. This distinctness was previously experimentally shown^7,9^ and is documented herein by ncSSR analysis. The SSR panel designed for the *Stub* genome was able to detect significant differences in studied loci, especially in telomeric parts. Another factor is the general chromosomal instability of dihaploids^36^. Since the dihaploid potato used for fusion was induced by *S. phureja*, a risk of occurrence of frequent *Stub*/*Sphu* DNA rearrangements^37^ affecting meiotic division can be expected. It is important to consider whether fertility could be improved using dihaploid potato derived from microspores. This is difficult to assess, because the latest published experiments with somatic hybrids^7^ also worked with *Sphu*-induced potato dihaploids and the experience with fertility was similar. Successful and fertile somatic hybrids were most likely obtained by the fusion of 4n *Stub* and 2n *Sblb* only^8,38^, when the predominating genome A likely enabled the hybrids to be fertile. The results obtained have supported the general recognition that with tighter ratios of incompatible genomes in somatic hybrids, problems arise with meiotic division as well as with the development of functional gametes.

Somaclonal variation is a phenomenon regarding breeding potential in potato and has been widely studied^15^. Naturally, an extension of variability in relatively uniform progenies can be detected in qualitative, quantitative, and molecular characteristics of tightly related somatic hybrids. The initiation of substantial genetic changes was related to the first three months, up until the regeneration of shoots from the callus. During long-term maintenance of plants from meristematic tissues (approximately 100 subcultures), the genotypes of somatic hybrids did not change. In the regeneration period, cells were exposed to the cytokinin zeatin, which can weaken the activity of enzymes responsible for repairing DNA^16^. This is supported by ncSSR analysis, since microsatellites alone are very susceptible to disfunctions of repairing enzymes^23^, in potato represented by *MSH2* gene. Mutations of the gene induces microsatellite instability^39^ but we probably can exclude this in relation to experience from long time maintenance. Detected genetic variations further agree with results of previous SSR^40^ and DArT^24^ analyses. The ncSSR panel presented here is informative in terms of reduced repair activity, but not as informative when regarding the identification of loci associated with phenotype differentiation. The phenotype variations supported by frequent molecular changes in DNA were the most substantial result of the study, with some complexes of mutations associated with specific phenotypes (ncSSR E genotype with inability to transfer ex vitro, A genotype with early date of emergence and flowering, and D genotype with high content of glycoalkaloids). Although genetic variations occurred in nuclear microsatellites, they were not observed in the cytoplasmic DNA. Plasmatic DNA was completely inherited from the *Stub* progenitor, which agrees with previous studies^41,42^ where the preferential inheritance of the *Stub* plastome was observed in several interspecific somatic hybrids, however Chandel et al*.*^43^ found significant differences just in cytoplasmic DNA. Considering the epigenetic background, molecular results indicated that the somaclonal variation in the progeny is well explainable by structural changes in DNA. However, epigenetic changes cannot be excluded completely because they have not been objectively studied here. Recent studies of somaclonal variation confirmed genetic changes in potato protoplast cultures^44^ and epigenetic (methylation) changes^45^ in clonally propagated potato. In contrast, study on epigenetics in coffee excluded these influences, and all somaclonal variation was explained by structural changes in DNA^46^. Another phenomenon to consider in our case is a somatic incompatibility^47^, which seems to be analogous to sexual incompatibility and incongruity. It is linked to chromosomal incompatibility, which reflects in many genetic rearrangements (and potentially in sterility of plants), and also to genome-plasmon interactions. Both usually prevent regeneration of plants because lead to discoordination of life cell cycle and metabolism. To synchronize all aspects to reach successful somatic hybrids is challenging, which is here documented by low ratio of regenerated plants to the current number of initiated microcalli.

The intermediate appearance of somatic hybrids in the majority of aboveground morphological traits and more intensive growth agreed with previous studies^48–50^. Additionally, the flower traits reported here, including shape arrangement and color of the flower corolla, are assumed to be controlled by several interacting genes, some of which have an additional pleiotropic effect on the color of stems, petioles, peels, or pulp of tubers. The adaptation of tubers induction on the short day and very late maturity agreed with the behavior of *S. bulbocastanum*^51^. The resistance against *P. infestans* was in accordance with previous observations of interspecific hybrids of *S. tuberosum* and *S. pinnatisectum*^52,53^ and interspecific hybrids of *S. tuberosum* and *S. cardiophyllum*^43^. Even though the expression of resistance was not as strong as in *Sblb*66, this study presents a high efficiency of the *Rpi-Blb-1* gene^10^ in the agri-environmental conditions of the Czech Republic.

The total content of glycoalkaloids in somatic hybrids was more comparable to *Stub* varieties. The low concentration of alkaloids found in *Sblb* was consistent with previous studies^54^. However, this contradicted the results by Savarese et al*.*^55^, who did not detect chaconine and solanine in *S. bulbocastanum*. Newly detected unknown glycoalkaloids in the hybrids presented here likely belong to the *Sblb* family of solanidine and solanidine-like alkaloids^56^. Their identification requires other analyses; a study of their ecological effects in interaction with the Colorado potato beetle could have potential. We observed that ncSSR genotype D significantly differed in the content of glycoalkaloids and that the characteristics associated with the loss of *Stub* allele 126 bp of the marker STI0014 was positioned on chromosome 09. This can indicate a hypothetical presence of some regulatory factors influencing the expression of glycoalkaloids on chromosome 09, which were neutralized by linked rearrangements near locus STI0014. However, an array of 10 genes that participate in glycoalkaloids biosynthesis were positioned^57^; six of them create a cluster on chromosome 07, and two are present in a duplicated region in chromosome 12. No other pathways linked to the glycoalkaloid biosynthesis have been reported.

Somaclonal variability is suitable for experimental research on genetic control of metabolic pathways^15^. The material presented here can be used in comparative studies of differences in transcriptomes and the expressional value of genes related to the synthesis of glycoalkaloids^58^ and dormancy, which can be influenced by the differential activity of specific cyclins with cyclin-dependent kinases^59^. Similarly, the activity of genes responsible for DNA repair, likely associated with homoeologous recombination^23^ and mismatch repair^39^, should be studied.

## Conclusions

The experimental conditions of protoplasts regeneration into plants were suitable for both production of somatic hybrids and a broadening of their genetic diversity. Detection of five different ncSSR genotype groups in hybrids proved a range of genetic changes which were responsible especially for significant differences in the total content of glycoalkaloids in foliage and in the date of emergence. Other less significant changes were observed in habitus and production characteristics of the plants. Although the somatic hybrids were highly resistant against late blight, they are out of any breeding potential being infertile. Instead, they will be used as “isogenic lines” for comparative analyses of transcriptomes in metabolic pathways linked to dormancy of tubers and biosynthesis of glycoalkaloids.

## Supplementary Information

Supplementary Figure 1.

Supplementary Figure 2.

Supplementary Tables 1-4.

Supplementary Tables 5-6.

Supplementary Table 7.

Supplementary Information 8.

## Data Availability

All allelic datasets are presented in Supplementary data (Tables S5 and S6) online. Unpresented datasets generated and analyzed during the current study are available from the corresponding author on reasonable request.
