# Supplementary figures and images for: Phenotypic, molecular and biochemical evaluation of somatic hybrids between Solanum tuberosum and S. bulbocastanum
Source: Sci Rep. 2022 Mar 16;12:4484. doi: 10.1038/s41598-022-08424-5 (PMC8927101; doi:10.1038/s41598-022-08424-5)

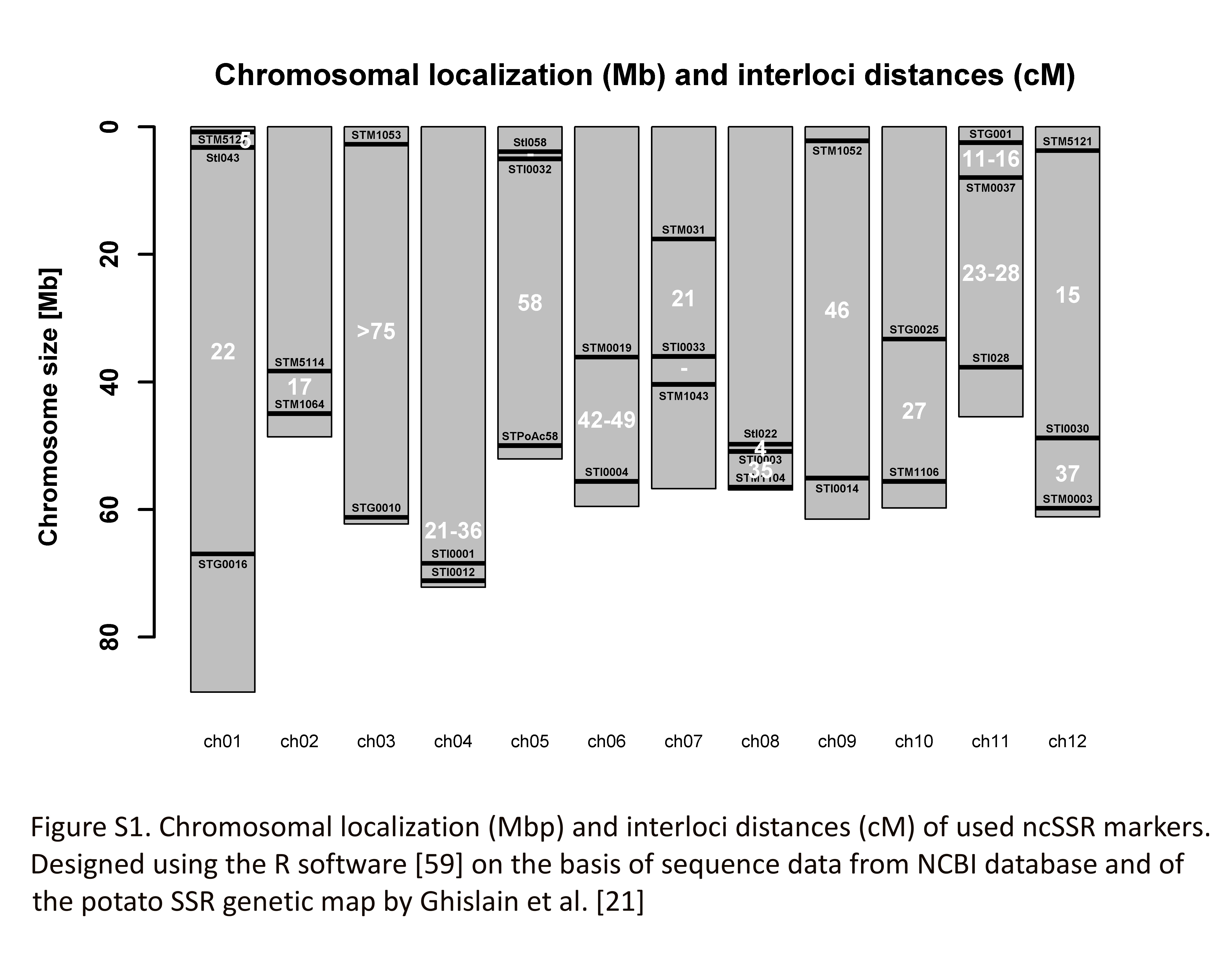

Supplement: Supplementary file 1 — Supplementary Figure 1. [file 41598_2022_8424_MOESM1_ESM.jpg]

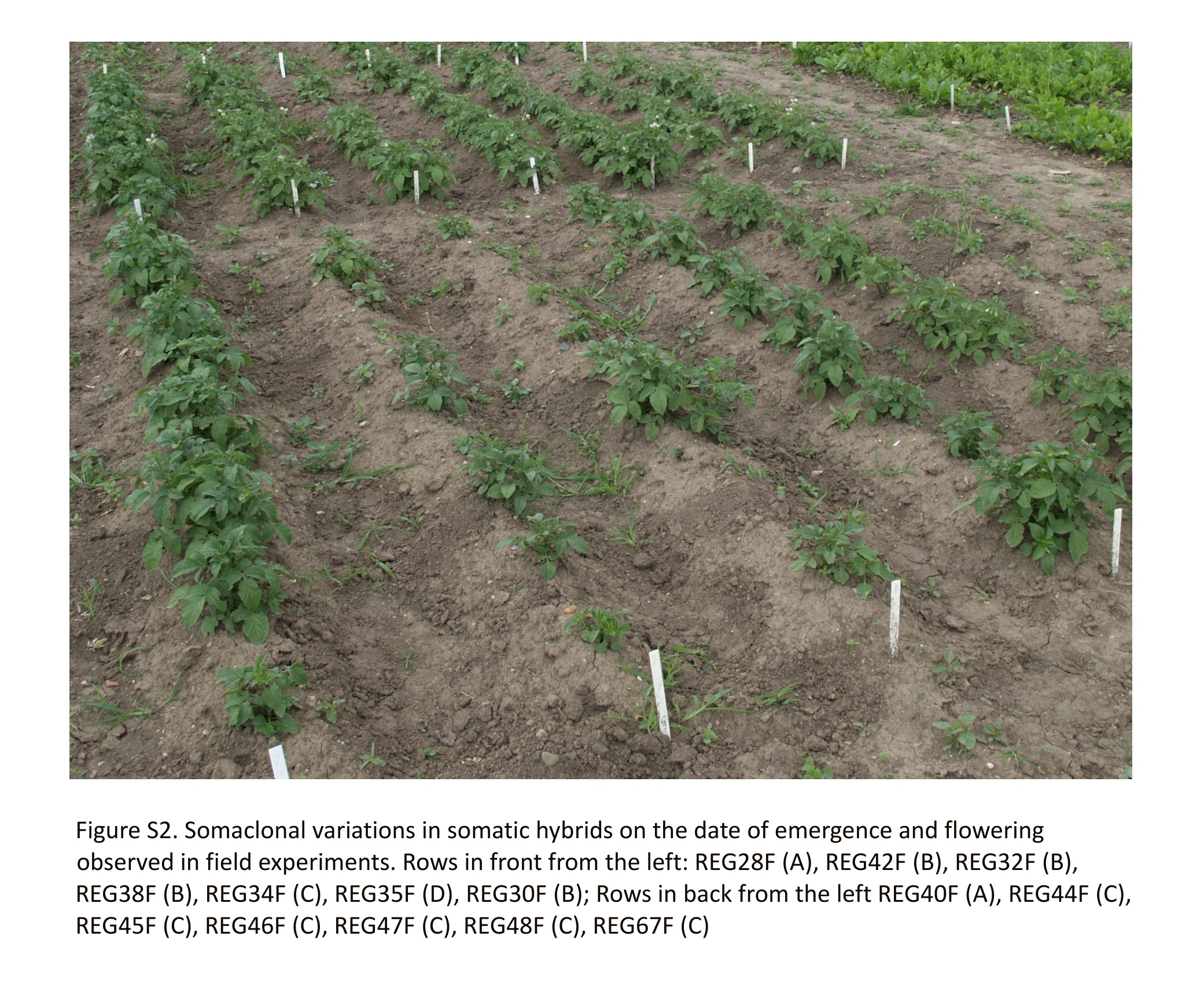

Supplement: Supplementary file 2 — Supplementary Figure 2. [file 41598_2022_8424_MOESM2_ESM.jpg]
