# Supplementary material for: Phenotypic, molecular and biochemical evaluation of somatic hybrids between Solanum tuberosum and S. bulbocastanum
Source: Sci Rep. 2022 Mar 16;12:4484. doi: 10.1038/s41598-022-08424-5 (PMC8927101; doi:10.1038/s41598-022-08424-5)
Supplement: Supplementary file 5 — Supplementary Table 7. [file 41598_2022_8424_MOESM5_ESM.docx]

**Table S7.** ANOVA table for quantitative parameters under effect of ncSSR genotype

| **Characteristics/Factor** | **Df** | **Sum Sq.** | **Mean Sq.** | **F-value** | **P-value** |
| --- | --- | --- | --- | --- | --- |
| **Characteristics of tubers** |  |  |  |  |  |
| *Number of tubers per plot* |  |  |  |  |  |
| ncSSR genotype | 4 | 5541.25 | 1847.08 | 1.75 | 0.189 |
| Residuals | 51 | 21089.37 | 1054.47 |  |  |
| *Weight of tubers per plot [kg]* |  |  |  |  |  |
| ncSSR genotype | 4 | 57.77 | 19.26 | 14.17 | 0.000 |
| Residuals | 51 | 27.18 | 1.36 |  |  |
| *Number of tubers over 3 cm per plot* |  |  |  |  |  |
| ncSSR genotype | 3 | 6381.54 | 2127.18 | 4.16 | 0.019 |
| Residuals | 20 | 10238.08 | 511.90 |  |  |
| *Weight of tubers over 3 cm per plot [kg]* |  |  |  |  |  |
| ncSSR genotype | 3 | 59.90 | 19.97 | 15.95 | 0.000 |
| Residuals | 20 | 25.04 | 1.25 |  |  |
| **Content of glycoalkaloids in dry matter** |  |  |  |  |  |
| *Chaconine in tubers [mg kg^-1^]* |  |  |  |  |  |
| ncSSR genotype | 4 | 557120.83 | 111424.17 | 3.59 | 0.007 |
| Residuals | 51 | 1643505.80 | 31009.54 |  |  |
| *Solanine in tubers [mg kg^-1^]* |  |  |  |  |  |
| ncSSR genotype | 4 | 522367.95 | 104473.59 | 2.57 | 0.037 |
| Residuals | 51 | 2153909.80 | 40639.81 |  |  |
| *Total glycoalkaloids in tubers [mg kg^-1^]* |  |  |  |  |  |
| ncSSR genotype | 4 | 2103858.52 | 420771.70 | 2.96 | 0.020 |
| Residuals | 51 | 7531268.42 | 142099.40 |  |  |
| *Chaconine in foliage [mg kg^-1^]* |  |  |  |  |  |
| ncSSR genotype | 4 | 21013279.29 | 4202655.86 | 32.32 | 0.000 |
| Residuals | 51 | 6890797.94 | 130015.06 |  |  |
| *Solanine in foliage [mg.kg^-1^]* |  |  |  |  |  |
| ncSSR genotype | 4 | 1804257.64 | 360851.53 | 31.58 | 0.000 |
| Residuals | 51 | 605682.30 | 11427.97 |  |  |
| *Total glycoalkaloids in foliage [mg.kg^-1^]* |  |  |  |  |  |
| ncSSR genotype | 4 | 34967055.11 | 6993411.02 | 32.28 | 0.000 |
| Residuals | 51 | 11481661.37 | 216635.12 |  |  |
