# Supplementary material for: Phenotypic, molecular and biochemical evaluation of somatic hybrids between Solanum tuberosum and S. bulbocastanum
Source: Sci Rep. 2022 Mar 16;12:4484. doi: 10.1038/s41598-022-08424-5 (PMC8927101; doi:10.1038/s41598-022-08424-5)
Supplement: Supplementary file 6 — Supplementary Information 8. [file 41598_2022_8424_MOESM6_ESM.docx]

**Supplementary file S8.** Supplementary information to methodology of phenotype evaluation

***Field trials and evaluation of morphology and pollen viability***

All genotypes were maintained *in vitro* in cultivation box MLR-351H (SANYO, Japan) on semisolid MS medium at a photoperiod of 16 hours of light with a temperature of 22°C, and 8 hours in darkness with a temperature of 18°C. For field tests, the genotypes were transferred to the greenhouse and then into field conditions of the Demonstration and Experimental Field of FAFNR CZU Prague-Suchdol (latitude 50.1276850N, longitude 14.3737717E, 275 masl) in order to obtain a sufficient quantity of tubers for field trials conducted in two successive years. Ten tubers of each genotype were planted per row in three randomized repetitions. The trial was subjected to the natural late blight (*Phytophthora infestans*) infection.

All twenty-seven morphological aboveground characteristics and eight characteristics of tubers were evaluated by a nine-point scale using a descriptor list designed for field evaluation of *Solanum* germplasm [34]. Ten best-flowering somatic hybrids and referential clones (‘Apta’ and *Sblb66*) were tested on a viability of pollen grains according to Wang *et al.* [35]. The *Stub*DH165 was not used due to its poor growth ability in field conditions.

***Evaluation of resistance against late blight***

Laboratory resistance to late blight was tested using *P. infestans* isolate Valečov (2010) virulent to *S. demissum* genes (*R1 – R11*)*.* The method of detached leaves test in Petri dish [36] was used; the strain was provided by the Department of Crop Protection, FAFNR CZU. The field resistance to late blight, explained as an increase of percentage of foliar damage, was evaluated weekly from July to September in seven consecutive observations.

***Evaluation of glycoalkaloid content***

For all genotypes, the content of glycoalkaloids (α-chaconine and α-solanine) in foliage and tubers was estimated. Freeze-dried ground samples of foliage/tubers (0.25 g) were extracted in 10 mL of methanol for 10 min in an ultrasound bath, centrifuged and filtered into a 25 mL volumetric flask. After repeated extraction, the volume in the flask was corrected to 25 mL using methanol. Then, 1 mL of solution was 10-times diluted by distilled water and mixed well. Five mL of the solution were purified on Solid Phase Extraction (SPE) column and analyzed by HPLC-MS/MS (high-performance liquid chromatography with mass spectrometry) in a setup of Ultimate 3000 RS (Dionex, USA) and 3200 QTRAP (Applied Biosystems, USA). Individual glycoalkaloids were identified by their molecular ions and product spectrum and quantified in mg kg^-1^ of dry matter using external calibration. All extractions were prepared and measured in three independent replicates.

Refferences:

34. Vidner, J. *et al.* Klasifikátor *genus Solanum* L. Výzkumný a šlechtitelský ústav bramborářský Havlíčkův Brod (Výzkumný ústav rostlinné výroby Praha - Ruzyně, 1987).

35. Wang, Z. Y., Ge, X., Scott, M. & Spangenberg, G. Viability and longevity of pollen from transgenic and non-transgenic tall fescue (*Festuca arundinacea*) (*Poaceae*) plants. *Am. J. Bot.* **91**(4), 523-530 (2004).

36. Sedlák, P. *et al.* Virulence and mating type of *Phytophthora infestans* isolates in the Czech Republic. *Scientia Agriculturae Bohemica* **48**(4), 185-192 (2017).
